# GAIT SPEED AND CHILDHOOD-TO-MIDLIFE BRAIN HEALTH: RETHINKING GAIT

**DOI:** 10.1093/geroni/igz038.3192

**Published:** 2019-11-08

**Authors:** Line Rasmussen, Avshalom Caspi, Terrie Moffitt, Harvey J Cohen, Miriam C Morey, Leah Richmond-Rakerd

**Affiliations:** 1 Dept. of Psychology and Neuroscience, Duke University, Durham, North Carolina, United States; 2 Duke University Medical Center, Durham, North Carolina, United States; 3 Durham Veterans Affairs Health Care System, Durham, North Carolina, United States

## Abstract

Background: Gait speed is a well-known predictor of functional decline and mortality in older adults, but little is known about the origins of gait speed earlier in life. We tested the hypothesis that slow gait reflects accelerated biological aging already at midlife, as well as poor neurocognitive functioning in childhood and childhood-to-midlife cognitive decline. Methods: Prospective study of the population-representative Dunedin Study birth cohort (n=1,037), followed to age 45 (until April 2019). We measured age-45 gait speed in 904 (90.7%) participants and tested associations with key life course factors. Results: The mean (SD) gait speeds (m/s) were: usual: 1.30 (0.17); dual task: 1.16 (0.23); and maximum: 1.99 (0.29). Among midlife adults, those with more physical limitations (β -0.27; P<.001), poorer physical functions (β 0.24–0.36; all P<.001), accelerated biological aging across multiple organ systems (β -0.33), older facial appearance (β -0.25), smaller brain volume (β 0.15), more cortical thinning (β 0.09), smaller cortical surface area (β 0.13), and more white matter hyperintensities (β -0.09) had slower gait speed, all P<.05. Participants with lower IQ in childhood (β 0.34) and midlife (β 0.38) and who exhibited childhood-to-midlife cognitive decline (β 0.10) had slower gait speed at midlife, all P<.01. Adults with poorer neurocognitive function as early as age 3 had slower gait in midlife (β 0.26; P<.001). Conclusion: Adults’ gait speed is more than an indicator of geriatric functional status, it is also an index of midlife aging and lifelong brain health.

